# Supplementary material for: Simultaneous trimodal single-cell measurement of transcripts, epitopes, and chromatin accessibility using TEA-seq
Source: eLife. 2021 Apr 9;10:e63632. doi: 10.7554/eLife.63632 (PMC8034981; doi:10.7554/eLife.63632)
Supplement: Transparent reporting form [file elife-63632-transrepform.docx]

***eLife’s* transparent reporting form**

We encourage authors to provide detailed information *within their submission* to facilitate the interpretation and replication of experiments. Authors can upload supporting documentation to indicate the use of appropriate reporting guidelines for health-related research (see [EQUATOR Network](http://www.equator-network.org/%20)), life science research (see the [BioSharing Information Resource](https://biosharing.org/)), or the [ARRIVE guidelines](http://www.plosbiology.org/article/info:doi/10.1371/journal.pbio.1000412) for reporting work involving animal research. Where applicable, authors should refer to any relevant reporting standards documents in this form.

If you have any questions, please consult our Journal Policies and/or contact us: [editorial@elifesciences.org](mailto:editorial@elifesciences.org).

**Sample-size estimation**

- You should state whether an appropriate sample size was computed when the study was being designed
- You should state the statistical method of sample size computation and any required assumptions
- If no explicit power analysis was used, you should describe how you decided what sample (replicate) size (number) to use

Please outline where this information can be found within the submission (e.g., sections or figure legends), or explain why this information doesn’t apply to your submission:

No sample-size estimation was performed for this study.

**Replicates**

- You should report how often each experiment was performed
- You should include a definition of biological versus technical replication
- The data obtained should be provided and sufficient information should be provided to indicate the number of independent biological and/or technical replicates
- If you encountered any outliers, you should describe how these were handled
- Criteria for exclusion/inclusion of data should be clearly stated
- High-throughput sequence data should be uploaded before submission, with a private link for reviewers provided (these are available from both GEO and ArrayExpress)

Please outline where this information can be found within the submission (e.g., sections or figure legends), or explain why this information doesn’t apply to your submission:

Due to the expense involved in performing single-cell experiments, replicates were not used for most experiments. Where multiple technical replicates (e.g. multiple 10x Genomics Chromium wells) were used, they are described in the text. Criteria for inclusion/exclusion of individual cells based on quality metrics are described in the Results and Methods sections. HTS data is deposited or in the process of deposit (dbGAP) as described in the Data and Code Availability section of the manuscript.

**Statistical reporting**

- Statistical analysis methods should be described and justified
- Raw data should be presented in figures whenever informative to do so (typically when N per group is less than 10)
- For each experiment, you should identify the statistical tests used, exact values of N, definitions of center, methods of multiple test correction, and dispersion and precision measures (e.g., mean, median, SD, SEM, confidence intervals; and, for the major substantive results, a measure of effect size (e.g., Pearson's r, Cohen's d)
- Report exact p-values wherever possible alongside the summary statistics and 95% confidence intervals. These should be reported for all key questions and not only when the p-value is less than 0.05.

Please outline where this information can be found within the submission (e.g., sections or figure legends), or explain why this information doesn’t apply to your submission:

Figures 1 and 2, the number of cells used are presented in Figure 1b and Table 1.

Figure 1f: definitions of center and error bar parameters are described in the figure legend.

Figure 2e, f: Details for statistical tests of enrichment are presented in Methods.

Figure 3: Number of cells are displayed in the figure legend for panel a.

Figure 4: Number of cells displayed in panels are provided in figure legends.

(For large datasets, or papers with a very large number of statistical tests, you may upload a single table file with tests, Ns, etc., with reference to sections in the manuscript.)

**Group allocation**

- Indicate how samples were allocated into experimental groups (in the case of clinical studies, please specify allocation to treatment method); if randomization was used, please also state if restricted randomization was applied
- Indicate if masking was used during group allocation, data collection and/or data analysis

Please outline where this information can be found within the submission (e.g., sections or figure legends), or explain why this information doesn’t apply to your submission:

Group allocation was not performed for this study.

**Additional data files (“source data”)**

- We encourage you to upload relevant additional data files, such as numerical data that are represented as a graph in a figure, or as a summary table
- Where provided, these should be in the most useful format, and they can be uploaded as “Source data” files linked to a main figure or table
- Include model definition files including the full list of parameters used
- Include code used for data analysis (e.g., R, MatLab)
- Avoid stating that data files are “available upon request”

Please indicate the figures or tables for which source data files have been provided:

Figure 1b, Top Row and Figure 1f: Figure-1_Source-Data-1_QC-Metrics.csv

Figure 1b, Bottom Row: Figure-1_Source-Data-2_Fragment-Size-Distributions.csv

Figure 1c: Figure-1_Source-Data-3_TSS-Footprint-Pileups.csv

Figure 1d: Figure-1_Source-Data-4_CTCF-TSD-Center-Pileups.csv

Figure 1e: Figure-1_Source-Data-5_Read-Fraction-Summary.csv

Figure 2a-c: Figure-2_Source-Data-1_UMAP-Coords_Label-Scores.csv

Figure 2d: Figure-2_Source-Data-2_Cell-Type-Fractions.csv

Figure 3a-d and Figure 3f: Figure-3_Source-Data-1_Type-labels-UMAP-.csv

Figure 3e: Figure-3_Source-Data-2_ADT-UMI-Counts.csv

Figure 4b-d: Figure-4_Source-Data-1_Barcode-QC-Metrics.csv

Figure 4e-f: Figure-4_Source-Data-2_Type-Labels-UMAP.csv

Figure 4g-j: Figure-4_Source-Data-3_PeakLinks.csv

Code for analysis will be deposited to Github, as described in the Data and Code Availability section.
